# Noninvasive Measurement of [^11^C]PiB Distribution Volume Using Integrated PET/MRI

**DOI:** 10.3390/diagnostics10120993

**Published:** 2020-11-24

**Authors:** Hidehiko Okazawa, Masamichi Ikawa, Tetsuya Tsujikawa, Akira Makino, Tetsuya Mori, Yasushi Kiyono, Hirotaka Kosaka

**Affiliations:** 1 Biomedical Imaging Research Center, University of Fukui, Eiheiji-cho 910-1193, Japan; iqw@u-fukui.ac.jp (M.I.); awaji@u-fukui.ac.jp (T.T.); amakino@u-fukui.ac.jp (A.M.); morit@u-fukui.ac.jp (T.M.); ykiyono@u-fukui.ac.jp (Y.K.); 2 Department of Advanced Medicine for Community Healthcare, Faculty of Medical Sciences, University of Fukui, Fukui 910-1193, Japan; 3 Department of Neuropsychiatry, Faculty of Medical Sciences, University of Fukui, Fukui 910-1193, Japan; hirotaka@u-fukui.ac.jp

**Keywords:** PET/MRI, amyloid imaging, distribution volume, SUVR, quantitative analysis, IDIF

## Abstract

A noninvasive image-derived input function (IDIF) method using PET/MRI was applied to quantitative measurements of [^11^C] Pittsburgh compound-B (PiB) distribution volume (DV) and compared with other metrics. Fifty-three patients suspected of early dementia (71 ± 11 y) underwent 70 min [^11^C]PiB PET/MRI. Nineteen of them (68 ± 11 y) without head motion during the scan were enrolled in this study and compared with 16 age-matched healthy controls (CTL: 68 ± 11 y). The dynamic frames reconstructed from listmode PET data were used for DV calculation. IDIF with metabolite correction was applied to the Logan plot method, and DV was normalized into DV ratio (DVR) images using the cerebellar reference (DVR_L_). DVR and standardized uptake value ratio (SUVR) images were also calculated using the reference tissue graphical method (DVR_r_) and the 50–70 min static data with cerebellar reference, respectively. Cortical values were compared using the 3D-T1WI MRI segmentation. All patients were assigned to the early Alzheimer’s disease (eAD) group because of positive [^11^C]PiB accumulation. The correlations of regional values were better for DVR_L_ vs. DVR_r_ (r^2^ = 0.97) than for SUVR vs. DVR_r_ (r^2^ = 0.88). However, all metrics clearly differentiated eAD from CTL with appropriate thresholds. Noninvasive quantitative [^11^C]PiB PET/MRI measurement provided equivalent DVRs with the two methods. SUVR images showed acceptable results despite inferior variability and image quality to DVR images.

## 1. Introduction

Molecular imaging methods are used for the clinical diagnosis of neurodegenerative diseases based on visual delineation of functional or pathological changes in the brain. Recently, the amyloid and tau imaging technique using positron emission tomography (PET) has become widely used for the differential diagnosis of dementia and is considered to be important particularly for diagnosis of Alzheimer’s disease (AD) [1]. The accumulation reflects pathologic changes of aggregation of amyloid and neurofibrillary tangles caused by phosphorylated tau. Many simplified methods for evaluation of the accumulation intensity have also been developed. Several studies attempted to simplify quantitative PET measurements with [methyl-^11^C]-2-(4′-methyl-aminophenyl)-6-hydroxybenzothiazole ([^11^C]PiB) using the reference tissue graphical analysis method and the image-derived input function (IDIF) method [2,3,4,5], where accuracies were compared with quantitative measurements of distribution volume (DV) with arterial blood samples to estimate the true input function. In comparisons of these quantitative metrics and standardized uptake values (SUV) obtained from static images in the later phase, SUV ratio (SUVR) values normalized by cerebellar cortical counts, which is now usually used for the evaluation of cerebral tracer accumulation, correlated well with the DV ratio (DVR) in quantitative analysis [2,3,4,5].

The accuracy of the IDIF method for quantitative evaluation of various metrics in PET studies is controversial, and many correction methods such as partial volume (PVC) and spillover, and arterial or arterialized venous blood correction have been applied for [^11^C]PiB PET [2,5]. If the arterial input function (AIF) requires metabolite correction to estimate the real plasma input, the metabolite fraction or unchanged fraction of the tracer should be determined and applied to the whole blood IDIF. Mourik et al. (2009) compared three different brain PET tracers and concluded that [^11^C]PiB PET might require blood count calibration to adjust the radioactivity concentration to a real AIF. However, AIFs obtained from blood samples may be unreliable due to substantial errors caused by sticking of the tracer [5]. In such cases of highly lipophilic ligands, image-derived estimation of arterial radioactivity with appropriate correction methods including metabolite ratio may be more reliable than measurement with sampling blood in clinical studies.

We established a new calibration method using an integrated PET/MRI scanner to estimate precise arterial radioactivity from noninvasive IDIF [6]. In the present study, we applied our new IDIF method for estimation of the true input function without blood sampling, and compared regional values with DVR from graphical reference tissue method (DVR_r_) and SUVR. A high quality of IDIF estimation is an advantage of PET/MRI because of simultaneous acquisition of PET and MRI images in the same location and high reliability of IDIF counts by improvement of PET detectors [6,7]. To correct the metabolite fraction in the arterial blood, a Hill-type function was applied to the IDIF curves [5,8], eliminating the need for invasive arterial blood sampling in the deep PET/MRI gantry. We employed the segmentation method using individual anatomical images from MRI to differentiate the gray and white matters as well as cerebrospinal fluid (CSF) spaces. In this way, we could obtain precise regional values by reducing individual variability. The co-registration step of PET and MRI was eliminated by the simultaneous image acquisition. Using these new techniques, the thresholds of DVR and SUVR for diagnosis were determined and the diagnostic accuracies of these metrics were compared. DVR and other quantitative values have been compared between calculation methods of arterial input with metabolite correction and reference tissue method, and showed linear correlations [2,3,4,5,9]. SUVR is now widely used as a simple and easily obtained metric from a single static scan for 10–20 min in the later phase, while it has greater variation and substantial overlap between positive (AD) and negative (non-AD) patients [9,10]. We compared these metrics to evaluate feasibility of the new IDIF method using integrated PET/MRI.

## 2. Materials and Methods

### 2.1. Subjects

Fifty-three patients with decline of cognitive function (26 males and 27 females, 71 ± 11 y) who were diagnosed as mild cognitive impairment (MCI) or early Alzheimer’s-type dementia (DAT) were enrolled in a [^11^C]PiB PET/MRI study to evaluate cerebral amyloid deposition for assessment of pathophysiologic changes in dementia. A 70 min dynamic PET/MRI scan was performed for all patients; however, 19 patients showed negative [^11^C]PiB accumulation and 15 patients could not keep the same head position for the whole scan time. Finally, 19 patients (9 males and 10 females, 68 ± 11 y) who completed the 70 min scan without head motion were included in the quantitative evaluation of amyloid deposition. We recruited 16 age-matched healthy volunteers (10 males and 6 females, 68 ± 11 y) as a control group (CTL). All participants were interviewed to evaluate their cognitive ability using the Mini Mental State Examination (MMSE), and their cognitive ability has not declined for more than two years since the study. The study was approved by the Ethics Committee of the University of Fukui, Faculty of Medical Sciences (study protocol # 20170225, 25 February 2017), based on its guidelines (Ethical Guidelines for Medical Science Research with Humans) as well as the Helsinki Declaration of 1975 (revised in 1983). Written informed consent was obtained from each patient.

### 2.2. PET/MRI Scanner

A whole-body PET/MRI scanner (Signa PET/MR, ver. 26, GE Healthcare, Milwaukee, WI, USA) was used for simultaneous PET and MRI data acquisition [6,11]. The scanner permits PET acquisition of 89 image slices in 3D mode, with a slice thickness of 2.76 mm. Performance tests showed the intrinsic resolution of PET images to be 4.2–4.3 mm full width at half maximum (FWHM) in the transaxial direction. The PET/MRI scanner was calibrated beforehand with a dose-calibrator (CRC-12, Capintec Inc., NJ, USA) using a pool phantom and ^18^F-solution, according to the scanner manufacturer’s guidelines [6,7].

### 2.3. PET and MRI Image Acquisition and PET Reconstruction

The patients underwent brain PET/MRI scans using a standard head coil (8-channnel HD Brain, GE Healthcare) for simultaneous PET and MRI acquisition [6,7]. A 70 min list-mode 3D PET scan in time-of-flight (TOF) acquisition was started at the time of a bolus tracer injection of 700–750 MBq [^11^C]PiB via the antecubital vein. During the PET scan, a 3D radial MR acquisition for the zero-echo time (ZTE) method in the axial direction was performed for attenuation correction (AC) of PET data with the following parameters: field of view (FOV) 264 mm, matrix 110 × 110 × 116, voxel size 2.4 × 2.4 × 2.4 mm^3^, flip angle 0.8°, number of excitations 4, bandwidth ±62.5 kHz, and acquisition time of 41 s [12,13]. In the ZTE-AC method, the following process was used to create the MR-AC map based on a previous study [12]. Briefly, pre-filtering and histogram-based normalization were followed by intensity-based segmentation of the head, bias correction, identification of voxels affected by partial volume effects, and segmentation of the sinus, bone, and cavity masks. A pseudo-CT map was then generated with bone tissue scaled linearly based on the ZTE intensity. This mapping was determined by fitting of registered CT and ZTE data in the bone density range. To convert the pseudo-CT into a MR-AC map, the images were re-sampled with a 60 × 60 × 25 cm^3^ field of view (FOV) in a 128 × 128 × 89 matrix, and finally re-scaled to 511 keV attenuation coefficients.

Dynamic PET images were reconstructed from the PET data using the 3D ordered subset expectation maximization (OSEM) method and point spread function modeling algorithm in 39 frames of 12 × 5 s, 6 × 10 s, 3 × 20 s, 4 × 30 s, 5 × 60 s, 4 × 5 min, and 4 × 10 min. The following OSEM parameter set was applied for PET image reconstruction: subset, 28; iteration, 3; transaxial post-gaussian filter cutoff, 3 mm in 256 mm FOV, and 2 × 2 mm^2^ pixel size. Decay of radioactivity in the dynamic PET data was corrected to the starting point of each scan [14].

3D TOF MR angiography (MRA) and other anatomical MR images such as T2-weighted (T2WI) and FLAIR were acquired in the same position during the PET scan. High-resolution 3D-T1-weighted (T1WI) anatomical MRI was also collected using the following parameters: repetition time = 8.5 ms; echo time = 3.2 ms; flip angle = 12°; FOV = 256 mm; 256 × 256 matrix; 136 slices; voxel dimension = 1.0 × 1.0 × 1.0 mm^3^ [6,14].

### 2.4. Distribution Volume and SUV Image Calculation

Arterial time–activity curves (TACs) were obtained from the dynamic [^11^C]PiB-PET data using the IDIF method described previously [14]. Details of the IDIF estimation from PET/MRI data and its calibration method are described elsewhere [6,7]. In brief, the average image of the initial phase (10–40 s) of dynamic PET data was used to extract voxels only inside the internal carotid artery (ICA), where the 30 most intensely radioactive voxels were selected as the volume of interest (VOI) mask at the region of the clinoid to ophthalmic segment of the internal carotid artery (ICA) [15]. Individual 3D TOF-MRA images were used to verify the location of the arterial VOI mask precisely on the ICA. Arterial TACs in total blood radioactivity concentration were obtained by applying the ICA VOI masks to the 70 min dynamic PET data [7]. Before estimating arterial TACs using the IDIF method, PET counts in two phases were determined appropriately, that is, the maximal count or average count for each slice in the VOI mask. In the first phase, average values of maximal counts obtained from 3 to 5 slices in the VOI mask for each frame were used in the initial 40–50 s. In the second phase, the maximal values of each slice mean in the VOI mask were also calculated for each frame up to 70 min, and these two phases were combined [6]. In order to estimate the true plasma input function with metabolite correction (*C_p_*) from the IDIF TAC, the unmetabolized fraction (*R*) was estimated using a Hill-type function as follows [5,8]:

*R(t)* = α·*t*^β^/(*t*^β^ + γ),

where *t* (min) is the time after the tracer injection and α, β, γ are constants determined by a nonlinear least squares fitting. To estimate optimal constants, the unchanged plasma [^11^C]PiB fractions reported in a couple of previous studies were applied [2,4].

DV images (mL/g) were calculated on a pixel-by-pixel basis from linear regression slopes of the Logan plot method using *C*_p_ [16,17]. The last 50–70 min data plots were used for DV slope calculation. The DV images were then converted to DVR images using the mean DV value of the cerebellar cortex as a reference [9]. The Logan’s reference tissue graphical method was also applied to DVR_r_ image calculation using a TAC of the cerebellar cortex as a reference [9,17,18].

SUV images were calculated from static images of the 50–70 min time frame of the list-mode PET data, that is, the average accumulation during this period. Each static image was corrected for the individual patient’s body weight (BW: kg) and injection dose (ID: MBq) as follows: SUV = (PET count concentration [kBq/mL])/(ID/BW) [14]. To compare [^11^C]PiB accumulation in the brain, each SUV image was normalized by the individual SUV mean of the cerebellar cortex and saved as the SUV ratio (SUVR) image [10].

### 2.5. Statistical Analyses

We used PMOD software (version 3.9; PMOD Technologies Ltd., Zurich, Switzerland) to compare the regional DVR and SUVR values between the early AD (eAD) and CTL groups. Image parcellation was performed in PMOD, and the brain regions were segmented into 78 brain regions using the individual 3D-T1WI MRI image data. Regional values of SUVR and DVRs of Logan plot (DVR_L_) and reference tissue graphical methods (DVR_r_) were classified into each lobe, basal ganglia, and cerebellum from the individual regions of interest (ROIs) determined by the 3D-T1WI segmentation. The locations of PET and MRI were exactly the same due to simultaneous image acquisition in the PET/MRI scanner without subject head motion during the scan. The head position was confirmed by two anatomical MRI images at the beginning and end of PET acquisition. Repeated measures analysis of variance (ANOVA) with a post hoc paired *t*-test was applied to analyze differences in the values of regional DVRs and SUVR between eAD and CTL. Correlations of regional DVRs and SUVR values were also evaluated for linearity by Pearson’s regression analysis. *p* < 0.05 was considered significant. To determine the optimal cutoff value for differentiating two groups of CTL and AD, receiver operating characteristic (ROC) curve analysis was applied to the three metrics.

A general linear model in Statistical Parametric Mapping (SPM12; http://www.fil.ion.ucl.ac.uk/spm/, Wellcome Trust Centre for Neuroimaging, London, UK) was used to evaluate differences between images of DVR_r_, DVR_L_, and SUVR for the eAD and CTL groups. The PET images were anatomically normalized to the Montreal Neurological Institute (MNI) space with a voxel size of 2 × 2 × 2 mm^3^ using individual 3D-T1WI images and the template provided by SPM12. After the normalized images were spatially smoothed with a 10 mm Gaussian filter for group comparisons, each image pertaining to z values was entered into SPM12 with two-sample *t*-tests. We applied statistical thresholds of voxel-wise *p* < 0.001 uncorrected and cluster-level *p* < 0.05 family-wise error (FWE) corrected, for multiple comparisons in the whole brain.

## 3. Results

All patients showed positive cortical [^11^C]PiB accumulation and were assigned to the eAD group, while volunteers in the CTL group showed negative accumulation. There were no differences in age range, mean age, and gender ratio between the two groups. The MMSE scores for cognitive status were significantly different between the eAD (23.6 ± 3.3) and CLT (28.6 ± 2.6) groups (*p* < 0.00001).

**Figure 1:**
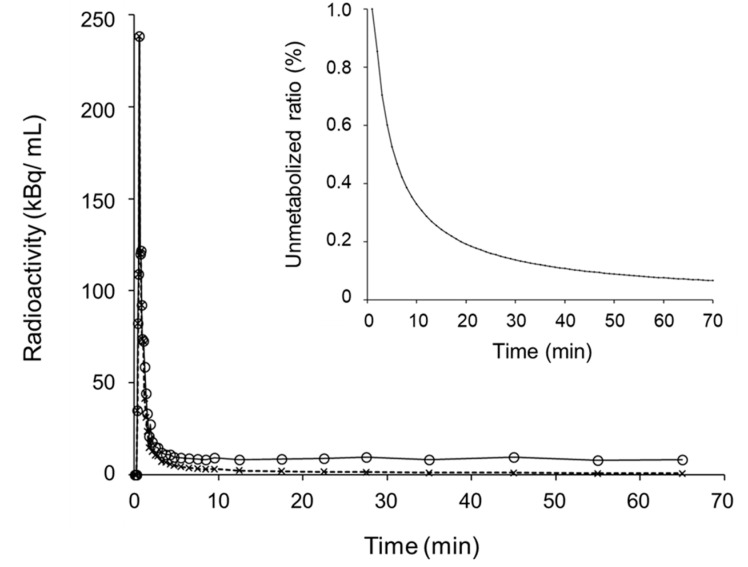
Representative time–activity curves (TAC) of the arterial blood (○) obtained from the image-derived input function (IDIF) method and metabolite-corrected plasma input (×) in a healthy control. Insert shows an arterial unmetabolized [^11^C]PiB ratio curve defined by a Hill-type function.

Figure 1 shows representative TACs of the arterial IDIF (solid line) and plasma input C_p_ corrected for the metabolite fraction (dashed line). The optimal parameters of the Hill-type function for C_p_ estimation were determined as α = 1.62, β = −0.92, and γ = 0.47 by a nonlinear least squares fitting. The insert graph of Figure 1 shows changes in the unmetabolized fraction as a function of time after the tracer injection. The TACs were almost the same for the IDIF and C_p_ in the initial frames, and gradually separated after the capillary phase.

**Figure 2:**
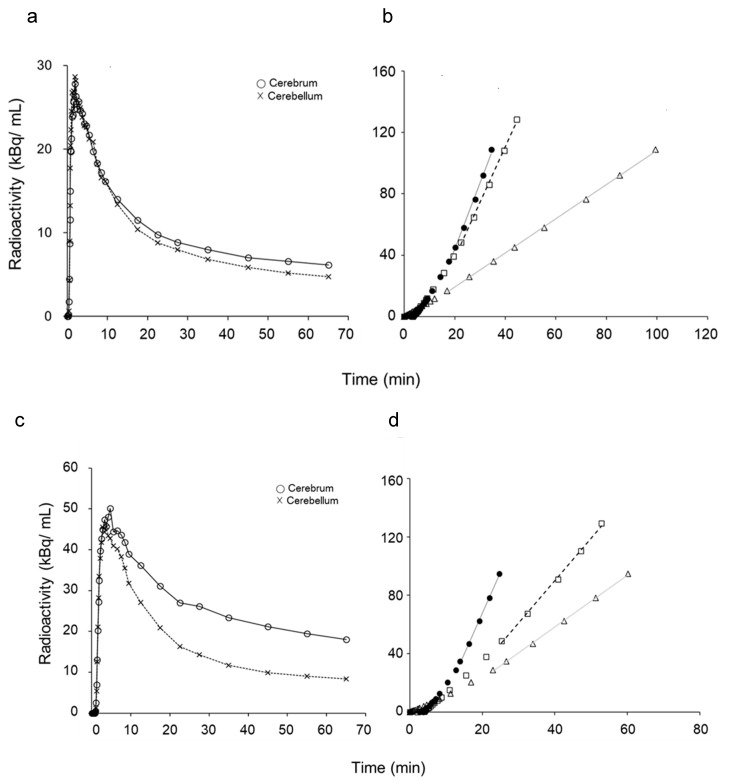
Representative cortical TACs of cerebrum (○) and cerebellum (×) obtained from dynamic PET data for each group of healthy control (CTL) (**a**) and early Alzheimer’s disease (eAD) subjects (**c**). Logan plots of [^11^C]PiB PET show good linear correlations in the later phases of a control (**b**) and an AD patient (**d**). The slopes of the regression lines were significantly different between cerebral cortex (● and solid line) and cerebellum (□ and dashed line) in AD patients (**d**), while they were similar in controls (**b**). The reference tissue graphical plot method also showed good linear correlations in the later phase (△ and gray line), but the slopes were completely different from the Logan plots because the cerebellar TACs were used for distribution volume ratio (DVR) calculation.

Representative TACs of the cerebral (solid line) and cerebellar (dashed line) cortices from each group are given in Figure 2. Logan plots for [^11^C]PiB PET showed good linear regressions in the later phases (black solid and dashed lines) in both eAD and CTL patients. The slopes of the regression lines were significantly different between the cerebral and cerebellar cortices in eAD patients (Figure 2d), while they were very close in controls (Figure 2b). The reference tissue graphical method based on the Logan plot method showed good linear correlations in the later phase (gray lines in Figure 2b,d); however, the slopes were completely different from the Logan plots because the slopes represent DV and DVR, respectively.

**Figure 3:**
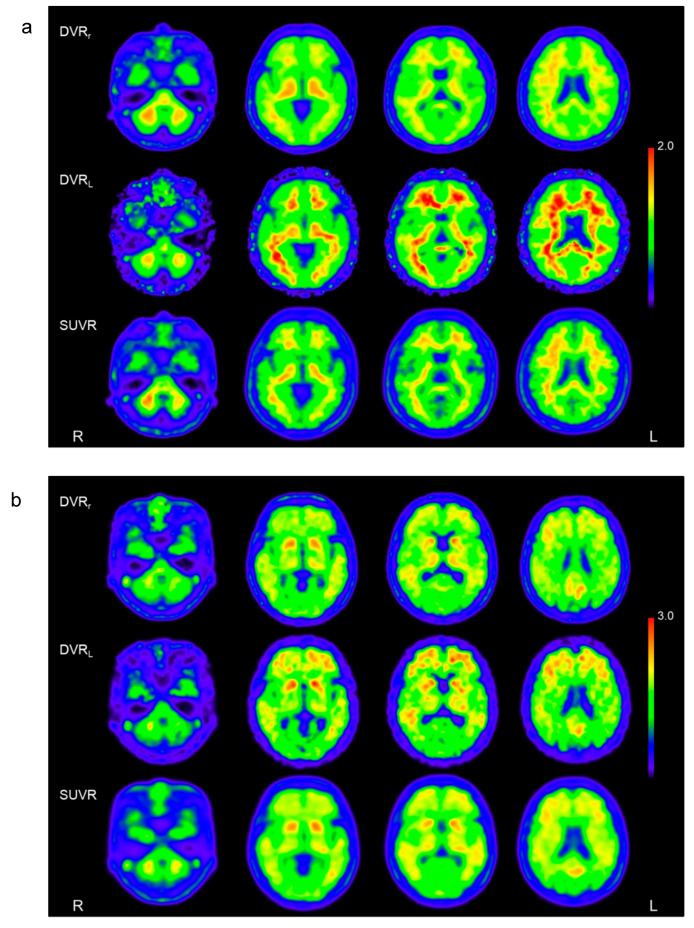
Representative images calculated from the [^11^C]PiB PET data of CTL (**a**) and eAD subjects (**b**). Iimages of distribution volume ratio calculated using the Logan plot method (DVR_L_) and metabolite-corrected IDIF (middle row) showed better contrast for both groups compared with distribution volume ratio from the reference tissue graphical method (DVR_r_) and standardized uptake value ratio (SUVR) (upper and bottom row, respectively).

Figure 3 shows representative images calculated from the [^11^C]PiB PET data of CTL and eAD subjects. DVR_L_ images showed better contrast for both subjects compared with DVR_r_ and SUVR. This tendency was observed in all subjects studied. Regional cortical values obtained from the anatomical segmentation of the brain are given in Table 1. All regional values were significantly greater in eAD subjects than in CTL. The values of the posterior cingulate cortex (PCC) were greater than other cortical regions, while the DVRs in the parietal lobe were not significantly different from those in the PCC.

**Figure 4:**
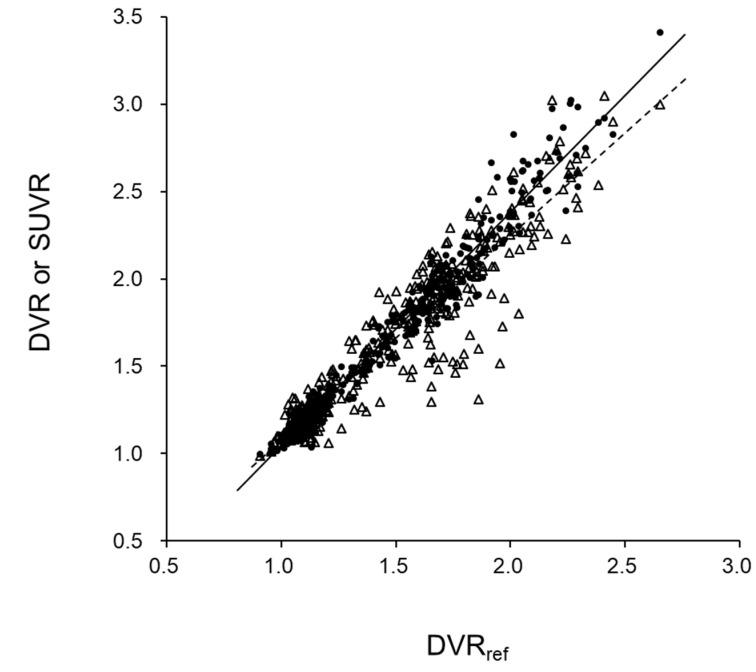
Regional values from all subjects were plotted to observe correlations between DVR_r_ vs. DVR_L_ (●) and DVR_r_ vs. SUVR (∆). DVR_r_ and DVR_L_ showed better correlation (solid line; y = 1.34x − 0.30, r^2^ = 0.97) than DVR_r_ and SUVR (dashed line; y = 1.17x − 0.09, r^2^ = 0.88).

**Figure 5:**
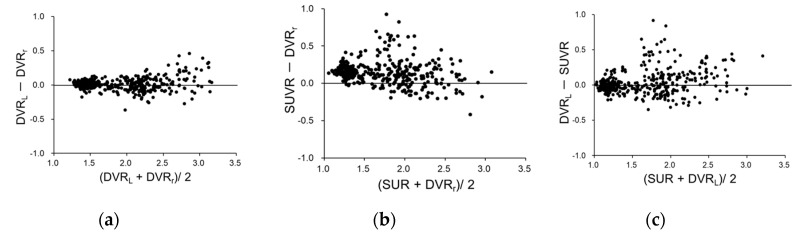
Balnd–Altman plots for DVR_r_ vs. DVR_L_ (**a**), DVR_r_ vs. SUVR (**b**), and DVR_L_ vs. SUVR (**c**) correlation. (**a**,**b**) are plots after correction with equations of linear regression.

Regional values from all subjects were plotted to observe correlations between DVR_r_ vs. DVR_L_ and DVR_r_ vs. SUVR (Figure 4). DVR_r_ and DVR_L_ showed a better correlation (r^2^ = 0.97) than that of DVR_r_ and SUVR (r^2^ = 0.88). The variance in correlation was greater for DVR_r_ vs. SUVR than for DVR_r_ vs. DVR_L_. However, SPM analysis did not show regional differences among the images of DVR_r_, DVR_L,_ and SUVR in each group of CTL and eAD subjects. The Balnd–Altman plots after correction with equations of linear regression for DVR_r_ vs. DVR_L_ (Figure 5a) and DVR_r_ vs. SUVR (Figure 5b) show significantly greater variance for the latter plot (*p* < 0.0001) than for the former. Figure 5c shows the Balnd–Altman plot for correlation between DVR_L_ vs. SUVR, which also shows a greater variance compared with DVR_r_ vs. DVR_L_ (Figure 5a).

**Figure 6:**
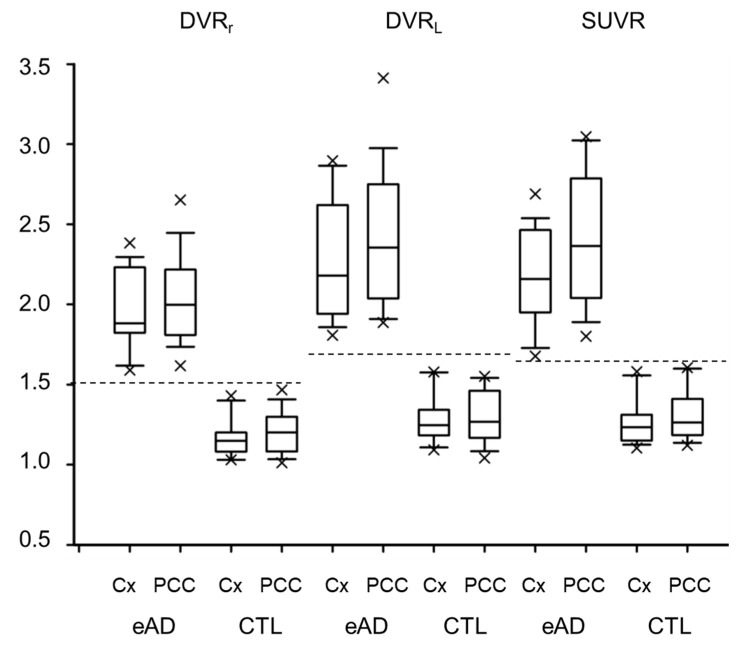
Cortical maximum values (Cx) of eAD and CTL obtained from each image. The posterior cingulate cortex (PCC) showed greater values than the cortical maximum among frontal, temporal, parietal, and occipital lobes. Dashed lines show optimal thresholds between eAD and CTL subjects. × is outlier of each group.

Figure 6 shows a comparison of the metric distributions for the eAD and CTL groups. Cortical maximum values, the highest cortical mean value among all regions of frontal, temporal, parietal and occipital lobes, and those from the PCC were compared among three different methods. The PCC tended to show greater values than the cortical maximum, although they were not significantly different. According to the ROC analysis, the optimal threshold values for differentiating eAD and CTL subjects were 1.5, 1.7, and 1.6 for DVR_r_, DVR_L_, and SUVR, respectively. These thresholds clearly differentiated eAD from CTL for all metrics. The area under the curves (AUC) values were >0.99 for DVR_r_ and DVR_L_, and 0.988 for SUVR in the ROC analysis.

## 4. Discussion

The present study evaluated feasibility of our new noninvasive IDIF method for the quantitative [^11^C]PiB PET/MRI study by comparing the results with those from the reference tissue graphical method as well as SUVR. We applied a Hill-type metabolite correction for estimation of the true input function (C_p_) in the IDIF, which was applied for DV image calculation in a couple of previous [^11^C]PiB studies [2,5]. All metrics were compared after normalization using the cerebellar cortical values as a reference, that is, DVR_L_ obtained from the DV image of the Logan plot, DVR_r_ calculated by the reference tissue graphical plot method, and SUVR. The correlation of regional DVR_L_ and DVR_r_ values was excellent, while SUVR showed a greater variance and a slightly inferior correlation coefficient compared with the two DVRs. Differences in metrics between the eAD and CTL groups were greater in the quantitative DVRs than in SUVR, suggesting that DVR images may provide better diagnostic performance than SUVR, although the two groups were differentiated well by the appropriate threshold for each metric determined in the present population. In the comparison of DVR_r_ and DVR_L_, the differences in cortical values between the two groups were greater and the image contrast was better in DVR_L_ than in DVR_r_. Thus, if a 70 min dynamic [^11^C]PiB PET/MRI scan is available, DVR_L_ image calculation with IDIF and the Hill-type correction seems to be a recommended method for quantitative analysis. However, if a patient cannot stay in the same position during a long dynamic scan, SUVR also provides reasonable results. In cases of borderline stage dementia, 70 min dynamic [^11^C]PiB PET/MRI scans and quantitative DVR image calculation are recommended.

Recently, the impact of amyloid and tau imaging has significantly increased in terms of pathologic evidence for differential diagnosis of eAD [1]. The molecular imaging based on such findings is considered to be more reliable than diagnosis from clinical symptoms or changes in cerebral energy metabolism, which have been used in the clinical setting for a long time, although recent advanced [^18^F]fluorodeoxy glucose (FDG)-PET studies showed similar diagnostic ability [19,20]. SUVR images in amyloid PET and their visual inspection may be sufficient for clinical diagnosis based on positive/negative discrimination according to the amyloid imaging guidelines or instructions [10]. However, for follow-up studies or multicenter studies, quantitative or semiquantitative indices are needed for comparisons of the severities and clinical stages of AD patients. In the present study, DVR_L_ showed the greatest difference among the three metrics between the eAD and CTL groups. Appropriate DVR calculation will provide a reliable index for longitudinal and multicenter comparisons.

Our previous studies established the IDIF method using a PET/MRI scanner for estimation of the arterial input of PET tracers [6,7,14]. However, the input functions should be corrected if the concentration of arterial blood radioactivity includes substantial metabolites of the tracer. Because several previous studies showed changes in the unmetabolized [^11^C]PiB fraction as a function of time measured by high-performance liquid chromatography (HPLC) or thin-layer chromatography (TLC) [2,4], we were able to assume the true input curves by applying a Hill-type function estimated from the data of those previous studies. Nonlinear least squares fitting estimated appropriate constants for the function, and the unmetabolised fraction curve estimated the true input well (Figure 1). Using this function, each IDIF was corrected to the true input function (C_p_), which provided an excellent Logan plot curve with good linearity in the last phase of 50–70 min after injection (Figure 2). There were no cases showing poor linearity of the plot in this phase.

We calculated DVR_r_ images using the Logan’s reference tissue graphical method [18]. The cerebellar cortical ROIs were easier to create than the ICA ROIs for IDIF, and the stable TACs could be applied for graphical plot image calculation. Good-quality quantitative images were available, although the contrast of the gray and white matters was slightly inferior to DVR_L_ calculated by IDIF (Figure 3). Regional DVR_r_ values were slightly lower than DVR_L_, which is similar to a previous result by Lopresti et al. [2]. In their study, three images calculated from arterial blood sampling with metabolite collection, IDIF, and SUVR showed similar regional values, but the reference tissue graphical method (DVR_r_) showed slightly lower regional values.

We did not sample arterial blood in the present study, which may be a limitation. However, estimating the plasma input function from arterial blood sampling and metabolite correction may not be an ideal method because the plasma counts and unmetabolized fraction obtained from HPLC or TLC may include substantial errors. Mourik et al. admitted the unreliability of plasma counts which were sometimes influenced by sticking of the [^11^C]PiB due to its high lipophilic property, and suggested that IDIFs might be more reliable [5]. The input functions obtained by arterial blood sampling and the IDIF method were not corrected for the dispersion effect of peripheral arteries in their study. The reference tissue graphical plot method is assumed to be corrected for all these possible biases of metabolites and dispersion, which could make it the most acceptable method for studies of lipophilic ligand kinetics if a reference region can be determined appropriately.

## 5. Conclusions

The noninvasive IDIF method for [^11^C]PiB PET/MRI provided quantitative DV and DVR with high-quality images. The accuracy of DVR_L_ was almost identical to that of DVR_r_, calculated by the reference tissue graphical plot method, with excellent correlation. SUVR images also showed acceptable results as a quantitative metric despite their greater variability compared with DVR images. If a long dynamic PET/MRI scan is available, quantitative DVR images calculated using the IDIF or a reference tissue TAC are recommended for patient follow-ups or multicenter studies.

## Figures and Tables

**Table 1:** Regional DVR (mL/min/100 g) and SUVR values (mean ± SD).

		DVR_r_		DVR_L_		SUVR	
		CTL	eAD	CTL	eAD	CTL	eAD
Frontal	L	1.10 ± 0.11	1.78 ± 0.23 ^†,^ *	1.22 ± 0.14	2.14 ± 0.32 ^†,^ *	1.20 ± 0.13	2.00 ± 0.30 ^†,^ *
	R	1.13 ± 0.10	1.79 ± 0.25 ^†,^ *	1.24 ± 0.13	2.17 ± 0.38 ^†^	1.23 ± 0.13	2.04 ± 0.32 ^†,^ *
Temporal	L	1.07 ± 0.05	1.52 ± 0.16 ^†,^ *	1.13 ± 0.06	1.72 ± 0.22 ^†,^ *	1.17 ± 0.08	1.78 ± 0.27 ^†,^ *
	R	1.07 ± 0.05	1.53 ± 0.16 ^†,^ *	1.13 ± 0.06	1.74 ± 0.23 ^†,^ *	1.16 ± 0.08	1.79 ± 0.28 ^†,^ *
Parietal	L	1.11 ± 0.11	1.87 ± 0.22^†,*^	1.18 ± 0.12	2.11 ± 0.31^†,*^	1.18 ± 0.12	2.06 ± 0.32^†,*^
	R	1.13 ± 0.11	1.91 ± 0.27 ^†^	1.19 ± 0.13	2.19 ± 0.38 ^†^	1.21 ± 0.14	2.10 ± 0.32 ^†,^ *
Occipital	L	1.10 ± 0.05	1.57 ± 0.19 ^†,^ *	1.16 ± 0.07	1.73 ± 0.25 ^†,^ *	1.16 ± 0.06	1.73 ± 0.28 ^†,^ *
	R	1.11 ± 0.06	1.60 ± 0.17 ^†,^ *	1.16 ± 0.08	1.78 ± 0.24 ^†,^ *	1.19 ± 0.08	1.76 ± 0.21 ^†,^ *
PCC		1.20 ± 0.13	2.04 ± 0.28 ^†^	1.30 ± 0.16	2.43 ± 0.42 ^†^	1.31 ± 0.16	2.42 ± 0.41 ^†^

CTL: healthy control, eAD: early Alzheimer’s disease, DVR_r_: DVR values calculated from reference tissue method using cerebellar cortex, DVR_L_: DVR from dynamic [^11^C]PiB PET data and Logan plot, SUVR: standardized uptake value ratio. ^†^ *p* < 0.00001 in comparing eAD and CTL subjects, * *p* < 0.05 in comparison with PCC.
